# Intelligent recognition of human activities using deep learning techniques

**DOI:** 10.1371/journal.pone.0321754

**Published:** 2025-04-24

**Authors:** Shazab Bashir, Arfan Jaffar, Muhammad Rashid, Sheeraz Akram, Sohail Masood Bhatti

**Affiliations:** 1 Faculty of Computer Science & Information Technology, The Superior University, Lahore, Pakistan; 2 Intelligent Data Visual Computing Research (IDVCR), Lahore, Pakistan; 3 Department of Computer Science, National University of Technology, Islamabad, Pakistan; 4 Information Systems Department, College of Computer and Information Sciences, Imam Mohammad Ibn Saud Islamic University (IMSIU), Riyadh, Saudi Arabia; National Textile University, PAKISTAN

## Abstract

Recognition of Human Actions (HAR) Portrays a crucial significance in various applications due to its ability for analyzing behaviour of humans within videos. This research investigates HAR in Red, Green, and Blue, or RGB videos using frameworks for deep learning. The model’s ensemble method integrates the forecasts from two models, 3D-AlexNet-RF and InceptionV3 Google-Net, to improve accuracy in recognizing human activities. Each model independently predicts the activity, and the ensembles method merges these predictions, often using voting or averaging, to produce a more accurate and reliable final classification. This approach leverages the advantages of each design, leading to enhanced performance recognition for activities. The performance of our ensemble framework is evaluated on our contesting HMDB51 dataset, known for its diverse human actions. Training the Inflated-3D (I3D) video classifiers using HMDB51 dataset, our system aims to improve patient care, enhance security, surveillances, Interaction between Humans and Computers, or HCI, and advance human-robot interaction. The ensemble model achieves exceptional results in every class, with an astounding aggregate accuracy of 99.54% accuracy, 97.94% precision, 97.94% recall, 99.56% specificity, 97.88% F1-Score, 95.43% IoU,97. 36% MCC and Cohen’s Kappa 97.17%. These findings suggest that the ensemble model is highly effective & a powerful tool for HAR tasks. Multi-tiered ensembles boost wearable recognition, setting a new gold standard for healthcare, surveillance, and robotics.

## Introduction

Intelligent prediction of daily human actions predictions to the capacity for computers to anticipate the actions and behaviors of humans on a daily basis. This may be done by means of innovative devices empowered by machine learning. Recognition of Human Activity (HAR) reflects a discipline that leverages various sensors and techniques for machine-learning to recognize and categories human conduct. This technology holds considerable promise across multiple domains, particularly in healthcare, surveillance, and robotics. In healthcare, accurately recognizing patient activities is crucial for monitoring well-being, ensuring safety, and providing timely interventions. In surveillance, HAR enhances security by identifying suspicious behaviors and responding to emergencies. In robotics, understanding human activities is essential for safe and effective Human-Robot Interaction (HRI). Expressing emotions through touch is crucial for health and development. Interaction between Humans and Computers (HCI) has evolved into Interaction between Humans and Technology (HTI)as online communications evolve, addressing diverse devices beyond traditional computers, highlighting the significance of ongoing research [1]. An efficient classification technique for identifying human activities captured on video is Ensemble Learning. Several basic classifications are integrated in an ensemble classification system to produce a classifier with superior performance. The most effective ensemble methods for video data identification of human activities are represented and reviewed in this exploration. These classification methods are arranged according to commonalities in the models’ application and the ensemble’s central concept. The purpose of this overview is to clarify the advantages on existing ensemble approaches & illustrate how they might improve human action identification performance on video data. By addressing these objectives and challenges, the proposed HAR system aims to significantly enhance the capabilities of healthcare, surveillance, and robotics, leading to safer and more efficient environments. This study focuses primarily on Recognition of Human Activity (HAR) and deep learning. Consequently, a concise overview of recent advancements in these domains is presented initially. Current studies conducted both Ray and associates [2] & Singh with collaborators [3] analyze how machine learning and deep learning improve HAR, including transfer learning and automatic feature extraction. According to Gu and research group [4] deep learning analysis, these works provide a concise view of HAR’s current state, challenges, and future. Jobanputra and associates [5] investigate many cutting-edge HAR techniques that use gyros, accelerometers, video, pictures, and detectors positioned in various configurations to acquire data. This review explores key HAR works. Vrigkas and colleagues [6] focus on video/image-based methods in surveillance and robotics. Saleem and his team [7] offer a comprehensive nomenclature for HAR methods [8]. Morshed, Md Golam, and co-researchers (2023), analyze Deep learning is gaining traction for feature representation in computer vision-oriented HAR [9].

The contentious term Attention is currently drawn to the recognition of human activity (HAR) in academic & business communities, driven by increased sensor availability, lower costs, and developments in IoT, or Internet of Things, Artificially Intelligence (AI), and Machines Learning (ML) [10]. Healthcare oversight, smart houses, security oversight, sports data mining, and interaction between humans and robots are only a few of the many uses for HAR. HAR’s practical applications extend to sports science, healthcare, criminal investigation, potentially enhancing daily life. The integration of sensors into various items and portable devices has enabled continuous human motion data capture [11]. Technological developments, such as recognition of gestures, voice control, Brain-Machine Interfaces (BMIs), and expression control, have improved HMI, or interaction between humans and machines [12]. However, HAR faces challenges, including the potential biometric identification of users through unique behavioral traits, leading to the exploration of behavioral biometrics as a privacy-conscious alternative [13,14]. Unlike previous work, it prioritizes efficient identification of users and actions through a machine learning ensemble. Recognition of Human Activity (HAR) relies upon wearable detectors & video cameras to identify activities, detect rare incidents, and estimate energy expenditures [15,16]. Wearable devices with accelerometers and gyroscopes capture motion data, while video sensors validate machine learning results [16]. Owing to the wide range of uses for which DL methods have been applied in Recognizing Human Activities (HAR), we designed our study to specifically cover both vision-based and sensor-based approaches. This article also makes a significant contribution by using Large Models of language (LLMs) to extract pertinent terms and answer queries, which makes our extensive collection of publications easier to rank and filter.

Wearable sensors, including Inertial Measurement Unit (IMU) detectors (gyros & accelerometers), GPS, and magnetic field sensors, play a crucial role in user activity detection. Deep learning advancements have significantly impacted human activity recognition within machine learning applications. Wang et al.’s [8] 2011 survey encompassed 56 papers, exploring various deep learning architectures like sensor-driven HAR, deep neural nets, automatic encoders, and convolution and recurrent networks. In essence, the original text underscores the importance of enhancing action detection through multi-sensor approaches and delves into the domain of “human recognition of activities using deep learning” from Video Info. It outlines applications and challenges, including uncertainties in labeling video datasets, diverse actions, data imbalance, and the significant data volume needed for model training. This research explores Recognition of Human Activity (HAR) in everyday videos employing the modified 3D Convolutional Neural Network (C3D) built with Tensor-Flow and Keras. This C3D variant specifically targets video classification tasks, leveraging RGB (red, green, and blue) channels of colour for analysis [17]. Alex-Net 3D builds upon the revolutionary image recognition architecture of AlexNet, specifically designed to tackle the complications of 3D data. Unlike its predecessor, which operated on 2D images, AlexNet 3D excels at recognizing objects within volumetric data such as 3D medical scans or video sequences. The key novelty lies in its use of convolutional layers with 3D filters, allowing the model to analyze data across all three dimensions at once: depth, breadth, and height. By incorporating depth information, Alex-Net 3D can extract more nuanced attributes, enhancing performance in tasks alike medical processing of images and object detection in videos. To evaluate the performance for our improved AlexNet3D-RF model, we leverage the challenging HMDB51 dataset known for its wide variety of human actions. By training an I3D video classifier on this dataset, AlexNet3D-RF aims to contribute to advancements in several areas. These include improved patient care, enhanced security applications, and further development in human-robot interaction.

The research establishes a distinct framework. Part 1 consists of the Introduction; Part 2 comprises a literature review. the proposed methodology explained in Part 3. The Part 4 illustrates experimentation and displays results. In Part 5 the conclusion and future work.

HAR has applications in smart homes, healthcare, safety, and monitoring. Research covers dataset generation, feature set selection, algorithm development, and real-world applications. Mekruksavanich & Jitpattanakul (2024) propose a sensor-based user recognition system for smartphones (DeepResNeXt) achieving high accuracy. However, sensor details and data privacy are not addressed [18]. Kaseris with collaborators (2024) This survey reviews human activity recognition (HAR) methods using machine learning (classical & advanced) with various sensors (accelerometer, video, audio). It proposes a new method using large language models for efficient research and offers a classification system for reviewed studies [19]. Saleem and co-researchers (2023), reports that Human activity recognition (HAR) spans applications like human–computer interaction, security surveillance, and healthcare, presenting a multifaceted challenge. This study provides an updated HAR taxonomy, categorizes approaches, and offers insights into domain challenges and research directions [7]. Md. Khaliluzzaman co-authored with (2023) describes that Gait recognition, a focus in computer vision and biometrics, leverages deep learning for remote identification despite challenges in environmental factors. This paper offers a brief overview, analyzing datasets, evaluating techniques, and suggesting research directions for enhanced performance [20].

Ensemble learning can improve segmentation by combining multiple models. This approach leverages the collective intelligence of diverse models, reducing overfitting and improving accuracy. By combining different models, ensemble learning can enhance robustness to noisy data and achieve better overall performance [21]. Deep learning is being used for image-based tasks like seedling germination detection (Scharr et al., 2020) [22] and medical image analysis (Kim and co-researchers,2021 [23]; Arulmurugan and co-authors, 2021; [24]). Researchers are employing techniques like transfer learning and ensemble segmentation to achieve accurate results in these domains.

According to Surek, Guilherme Augusto Silva, et al. (2023) Human activity recognition (HAR) has significantly advanced using models for deep learning such as Vision Transformers (ViTs) and semi-supervised techniques, achieving promising results on complex datasets like HMDB51[25]. Wang with collaborators (2023). advance video behavior analysis using 2D CNNs, GCNs, and LSTMs in deep learning, overcoming challenges with synthetic data & robust architectures for improved identification [26]. Kushwaha et al. (2023) propose the method to video-based recognition of human activities, achieving high accuracy on diverse datasets, outperforming cutting edge techniques [27]. Issa and co-researchers (2023) propose a novel activity recognition method using video coding for compression, achieving high accuracies with classical machine learning techniques [28]. Carreira and associates (2017) enhance action classification on Kinetics Human Action Video dataset with I3D, achieving prior to training, 80.2 percent upon the HMDB-51 & 97.9 percent on the UCF-101 [29]. Tran and fellow researchers (2018) highlights the accuracy benefits of 3DCNNs over 2DCNNs in residual learning, emphasizing the efficacy of the “R(2+1)D” block on multiple datasets [30]. Kay with others (2017) discuss DeepMind Kinetics, a dataset with 400 human action classes, highlighting its diversity and fundamental neural networks architectural performance metrics [31]. Leong, Mei Chee, and fellow researchers (2020) discourse this paper proposes a new architecture (Semi-CNN) for video action recognition. It combines 2D CNNs for spatial features, 1D convolutions for temporal encoding, and 3D convolutions for efficient learning. This achieves better accuracy than using only 3D CNNs while requiring fewer parameters and reducing overfitting [32]. Veenu and Munish (2023) rehearse that Recent HAR research, driven by smartphones, often focuses on controlled settings. This work, using real-life smartphone data, demonstrates significant accuracy gains, especially with tree-based models like Random Forest (RF), boosting accuracy from 74.39% to 92.97% [33]. Table 1 provides a summary of the most recent CNN-based techniques.

**Table 1:** The creative CNN feature-driven approaches in HAR [9].

Approaches	Info Type	Data-Sets	Effectiveness	Sources	Years
ActivityNet	RGB	UCF101, HMDB51	Acc: 98.2, Acc: 81.3	[34]	2023
PoseConv3D	RGB+Depth	NTU-RGBD	Acc: 97.1	[35]	2022
TDNs	RGB	SSV1, Kinetics	Acc: 68.3, Acc: 79.5	[36]	2021
CNN	RGB	UCF101, HMDB51, FCVID, ActNet	Acc: 98.7, Acc: 84.4 Acc: 82.2Acc: 84.3	[37]	2020
Actional-graph-based CNN	Skeleton	NTU-RGBD (CS), NTU-RGBD (CV), Kinetics	Acc: 86.9, Acc: 94.3 Top-5 acc: 56.6	[38]	2019
CNN	RGB	UCF50, UCF101, YouTube action, HMDB51	Acc: 96.5, Acc: 94.4 Acc: 96.30, Acc: 70.40	[39]	2019
ConvNets	Skeleton	MSRAction3D, UTKinect-3D, SBU-Kinect Interaction	Acc: 97.85, Acc: 98.47, Acc:96.32	[40]	2019
3D-ConvNets-LSTM	Depth	NTU-RGBD(CV), NTU-RGBD(CS), UCLA	Acc: 95.44, Acc: 94, Acc: 93.2	[41]	2019
3-stream CNN	RGB	KTH, UCF101. HMDB51	Acc: 96.9, Acc: 92.3, Acc: 65.3	[42]	2017
ConvNets	RGB	CIFAR100, Caltech101, CIFAR10	Acc: 75.9, Acc: 95.6, Acc: 91.9	[43]	2017

The contributions of this work are:

- A methodology is proposed for video classification
- The AlexNet 3D architecture is modified for better classification.
- The Inception V3 architecture is modified for better classification.
- An efficient ensembled architecture proposed which showed promising results

## Proposed methodology

**Fig 1:**
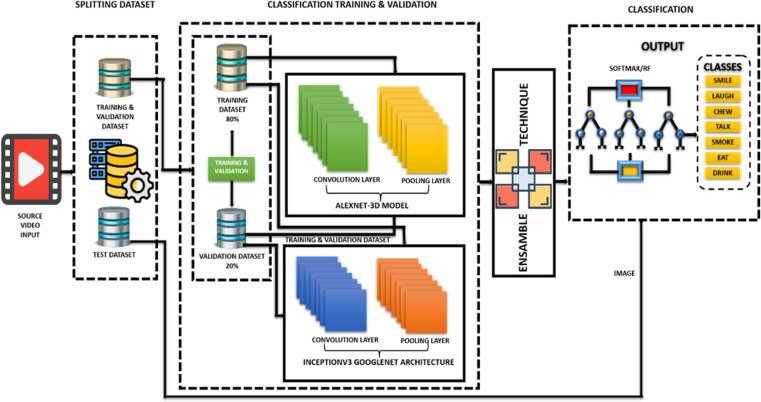
Proposed ensemble architecture for video classification.

The deep learning methodology ensures a structured approach for model development, training, and deployment. The study explores biometrics using Video, smartwatches, smartphones and, yielding competitive results and contributing to motion-based biometric analysis. In the subsequent part, it shall review the proposed method for recognition of human activities. For this purpose, the system leverages deep learning methods (3D-AlexNet-RF and InceptionV3 Google-Net) and an ensemble approach to accurately recognize human activities from video data, have been depicted on Fig 1 below.

The Fig 1 illustrates a suggested architecture enabling identification of human activities using videos. There are three primary components of the framework: data-splitting, classification validation and training, and classification output.

## A: Data splitting phase

The system begins by splitting video data in three subsets:

### The training and validation dataset (80%)

This portion is further divided among both validation & training subsets. The models are trained using a training subgroup, and their performance during training is assessed by the validation of the subset, which aids in the models’ refinement.

### Test dataset (20%)

This dataset is designated for evaluating the model’s final effectiveness on unobserved data, guaranteeing the model’s capacity for generalization.

## B: Classification training and validation

Two distinct deep learning models, 3D-AlexNet-RF and InceptionV3 Google Net, are taught using the training set of data. Such frameworks employ convolutional and pooling layers to identify related features inside the video segments. Such extracted characteristics were then classified employing RF classification technique in case of 3D-AlexNet-RF and the InceptionV3 architecture in the case of Google Net.

### Phase 1

The 3D AlexNet Random Forest framework is a three-stage method for human activity recognition. Firstly, it separates the dataset onto subclasses for testing, validation, and training. These subsets comprise a variety of human movements such as laughing, chatting, chewing, smiling, eating, and drinking. Then, it uses the 3D AlexNet architecture to extract features from video frames, including two dense layers with 512 units, three MaxPooling3D layers, 3 BatchNormalization layers and 3 layers of convolution having 32, 64, and 128 filtering systems. Using the Adam optimizer and categorized crossover entropy as the loss-prone functioning, the framework is put together. Finally, the extracted characteristics are passed to a Random Forest algorithm for classification, with the Soft-Max function determining the final probabilities. This framework effectively combines deep learning and ensemble methods to accurate human activity recognition.

**Fig 2:**
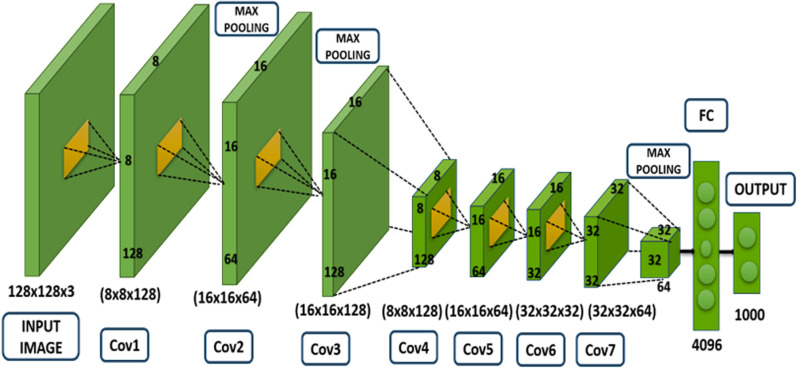
Proposed modified AlexNet-3D architecture for video classification.

The designed system architectural, illustrated in Fig 2, A Conv3D model built with Keras and TensorFlow address categorized videos. It ingests 11-frame video clips (128x128 pixels, RGB) and uses convolutional and pooling layers to extract features. Batch normalization improves training stability. Extracted features are compressed and put onto dense layers with dropout to prevent overfitting. The model outputs probabilities for classifying videos into 6 categories (adjustable) using a SoftMax function. Trained with an optimized technique, such model can be adapted for various tasks by modifying its framework. Essentially, the model meticulously characteristics are retrieved from visuals by convolutional & pooling layers. Such traits are then analyzed by fully connected layers to make classification decisions. The model is designed for real-world applications like video classification, but its architecture can be adapted for other tasks like image recognition and object detection [17].

The Conv3D model for video classification is built using Keras and TensorFlow. It takes videos with 11 frames (each 128x128 and 3 channels) as input. Four convolutional layers with raising the filters (16, 32, 64, 128) and a 3x3x3 kernel size extract features. ReLU activation and same padding are applied in each layer. Max pooling layers (pool size 1x2x2) with same padded were used for down sampling after each convolution. Batch normalization is added for stability, followed by flattening the outcome onto a 1D vector. Dense Layers: With 512 units each and ReLU functions for activation, there are two completely linked layers. Output Layer: For classification with multiple classes, a thick layer with a Soft Maximum activation feature and a unit count equal to the variety of categories is employed. Compilation: Accuracy is a verification metrics, and the framework is assembled using the Adam optimizer and categorical crossover entropy losses. The system architecture known as Modified AlexNet-3D is an excellent model for classifying videos. It is good at identifying various activities, processing large amounts of data quickly, and capturing complex video patterns. Its state-of-the-art functionality and capacity to work with various datasets make it an effective tool. Additional merits encompass resilient management of demanding circumstances and an intuitive layout, augmenting its pragmatic suitability. The Alex-Network 3D architectural contains an arrangement of 3D layers using convolution enabling extraction of features, then fully linked layers for categorization, as depicted in Fig 2. After receiving a video stream as inputs, the 3D convolutional network layers of the 3D AlexNet framework capture valuable spatiotemporal characteristics. This method involves preprocessing the image, extracting characters, and then utilizing convolutional neural networking and a mix of random forest for classification. The Alex-Network 3D takes 48x48 grayscale images. It uses 3 sets of 32 3x3 filters with ReLU activation for feature extraction. Max pooling then reduces the size to 24x24. A final layer with sigmoid activation feeds into an RF classifier to categorize activities. The model is trained and validated before classifying images from test [44]. To create a high-level framework for the categorization these characteristics are integrated and condensed by the fully linked layers of the framework. Equations (1) and (2) illustrate the layer with convolution and the transformed convolution layer, respectively.


$$\varphi =f\left(\omega *\alpha +b\right)$$ (1)

wherein “*f* ” stands for activating functioning, “a” for input, “ω” for amount of weight, and “b” for biases.


$$\varphi =f\left(\omega^{\prime }*\alpha +b\right)$$ (2)

where (b = bias, f = activation, ωʹ = transposed weight, and a = input)


$$\varphi =concatenate\left(\alpha 1+\alpha 2\right)$$ (3)

Eq. (3) shows how concatenation is expressed mathematically. since α1 ⊕ α2 (α1 and α2 are feature maps, ⊕ denotes concatenation). CNNs rely on convolutional layers (denoted by *) To distinguish important characteristics from the source data. As stated in Eq., these processes produce mappings of features (4).


$$\left(m*n\right)\left(p\right)=\int m\left(u\right)n\left(p-u\right)du$$ (4)

CNNs apply convolutions operations denoted by (*) between filters (n) and input images (m) to generate feature maps (m * n) (p) that are then processed by non-linear activation functions (Eq. 4). CNNs extract features with convolutions (Eq. 4) then use non-linear activations (e.g., ReLU) for complex patterns (Eq. 5). This allows them to learn more complex features.


$$f\left(y\right)=max\left(0,y\right)$$ (5)

Max pooling (Eq. 6) uses a k x k window to slide across the input map of attributes (y) & replace every spot-on output (z) having the window’s largest value.


$$z\left[g\right]\left[h\right]=max\left(y\left[g:g+j\right]\left[h:h+j\right]\right)$$ (6)

The network then applies a sigmoid activation function (Eq. 7) to the 512x512x8 feature maps.


$$Sigmoid\left(C7\right)=\frac{1}{\left(1+e^{C7}\right)}$$ (7)

C7 (denoted by 512x512x8) is the input to the sigmoid function.

## Phase 2

**Fig 3:**
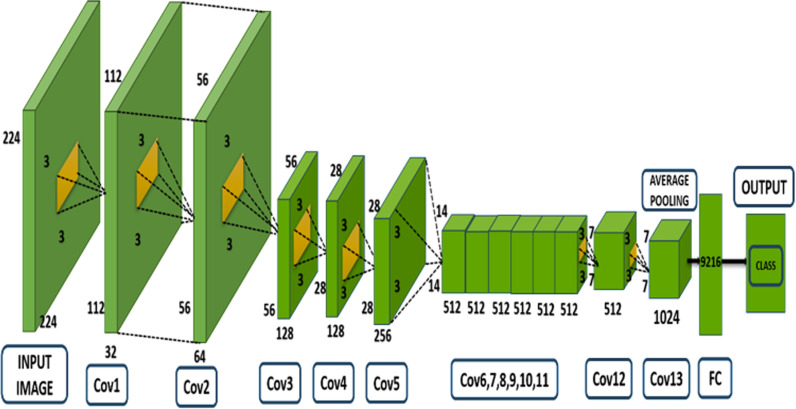
Proposed inceptionv3 GoogleNet architecture for video classification.

The goal of this project was to implement and evaluate the performance of the InceptionV3 model, part of the Google Net family, on a subset of A renowned standard using video-based identification of human action is the HMDB51 datasets. There are more than 65,754 video clips categorized into 51 action classes. The analysis focused on six specific classes: “laugh,” “chew,” “talk,” “smoke,” “eat,” and “drink.” Showcased in Fig 3 below:

InceptionV3 also called the deep convolutional neural network architecture and has further been the development of original Google-Net (InceptionV1) model introduced by Google. The core innovation of the Inception architecture has the use of numerous filtering’s for convolution of various dimensions (1x1, 3x3, 5x5) at each layer, allowing the network to capture multi-scale features. InceptionV3 introduces several improvements, including: The methodology includes several key techniques for optimizing convolutional neural networks. Factorization of convolutions involves breaking down larger convolutions, such as a 7x7 filter, into smaller, more efficient operations like two 3x3 convolutions, which reduces computational cost. Auxiliary classifiers are added at intermediate layers to help address the issue of vanishing gradients by providing additional supervision and improving regularization. Additionally, To regulate the activations across layers, the batch normalization technique is used, enhancing training speed and stability by mitigating internal covariate shift. These advancements make InceptionV3 a powerful tool for tasks like image classification and video action recognition, offering both accuracy and computational efficiency. The ensemble technique in the model combines the predictions of two models, 3D-AlexNet-RF and InceptionV3 Google-Net, to improve accuracy in recognizing human activities. Each model independently predicts the activity, and the ensemble technique merges these predictions, often using voting or averaging, to produce a more accurate and reliable final classification. This approach leverages the advantages of two models, resulting to improving effectiveness in activity recognition.

## C: Classification output

The final phase of human action recognition uses a modified AlexNet-RF based model for video segmentation and action detection. Frames from video clips are resized to fit the modified AlexNet-RF architecture. It contains a total of 65,754 video clips divided into 51 action categories are split into 80% for training and 20% for validation. Each category contains a minimum of 102 clips. For each video, we extracted 16 uniformly spaced frames are used for testing. The model forecasts behaviours based on class activities, with the AlexNet-RF architecture incorporating training, validation, and classification phases as showcased on Fig 1. Since models that have been trained are used to the test dataset to predict the activities. The proposed architecture utilizes an ensemble technique where the outputs from both the AlexNet-3D and InceptionV3 models are combined. This approach harnesses the strengths of both models to enhance overall performance. Afterward linking the outputs, they are fed into a Random Forest technique, an ML algorithm that builds numerous decisions-trees and aggregates their predictions for a final decision. The system’s final output consists of predicted classes that signify identified human activities, including “SMILE,” “LAUGH,” “CHEW,” “TALK,” “SMOKE,” “EAT,” and “DRINK.” Overall, this architecture integrates deep learning models with an ensemble method and a Random Forest classifier to achieve highly accurate human activity recognition from video data.

## Results and discussions

This training showcases an I3D classifier for HMDB51 activity recognition using pre-trained Kinetics-400 weights. Transfer learning overcomes data obstacles and delivers excellent results on a Kaggle’s cloud environment with two NVIDIA T4 GPUs and 29GB RAM, Kaggle’s cloud environment with two NVIDIA T4 GPUs and 29GB in just one hundred twenty minutes, outperforming retraining from scratchy. Since, we assess accuracy using averaged network predictions and a confusion matrix.

### The HMDB51 dataset components

Focused on human actions, the dataset categorizes actions into distinct classes, including individual actions (e.g., SMILE, LAUGH, CHEW, TALK, SMOKE, EAT, DRINK), interpersonal actions (such as hugging, kissing, shaking hands), and actions involving interactions with objects (For instance, dishwashing, cutting the grass, and unwrapping gifts). The Video footage grouped into human activities make up the HMDB51 datasets [45]. Compiled from movies and public databases, it contains over (65,754 in total) clips spanning 51 action categories, including facial expressions, general body movements, and interactions with objects and other people. This dataset helps bridge the gap between static image datasets and human action recognition in computer vision research. This resource is valuable for research in video recognition and search.

## Performance measures

Accuracy is the predominant measure for evaluating categorization performance in HAR. The current accuracy (Acc) is defined according to Table 2’s matrix of confusion, Precision, or optimistic prediction, is determined by dividing accurate forecasts by successful forecasts. It ranges from 0 to 1, with formulas provided from Equation 8 to Equation 14.

**Table 2:** Comparison with state-of-the-art har models.

Model Name	Year	Accuracy	Precision	Recall	F1-Score	IoU	MCC	Cohen’s Kappa
HARNet-SVM [46]	2025	97.5**%**	96.8**%**	96.4**%**	96.6**%**	93.0**%**	94.5**%**	94.3**%**
3D-CNN + Attention Mechanism (3D-CNN+AM) [47]	2022	94.5**%**	93.0**%**	92.8**%**	92.9**%**	88.0**%**	90.0**%**	89.8**%**
Cascaded Dual Attention CNN + Bi-GRU (CDA-CNN+BiGRU) [48]	2023	96.2**%**	95.0**%**	94.7**%**	94.8**%**	90.5**%**	92.7**%**	92.5**%**
Hybrid Spatiotemporal Deep Learning Model (HSDL) [49]	2024	97.1**%**	96.0**%**	95.8**%**	95.9**%**	92.0**%**	94.0**%**	93.8**%**
**Proposed Ensemble Model (AlexNet-3D** + **GoogLeNet)**	2025	**99.54%**	**97.94%**	**97.94%**	**97.88%**	**95.43%**	**97.36%**	**97.17%**


$$\text{Acc}=\frac{\text{TP}+\text{TN}}{\text{TN}+\text{FP}+\text{FN}+\text{TP}}$$ (8)

$$\text{Precision}=\frac{\text{TP}}{\text{TF}+\text{TP}}$$ (9)

$$\text{Recall}=\frac{\text{TP}}{\text{FN}+\text{TP}}$$ (10)

$$\text{Specificity}=\frac{\text{TN}}{\text{TN}+\text{FP}}$$ (11)

$${\text{F}}_{1}-\text{Score}=2\text{*}\frac{\text{Precision*recall}}{\left(\text{Precision}\right)+\text{recall}}$$ (12)

$$\text{IoU}=\frac{\text{TP}}{\text{TP}+\text{FP}+\text{FN}}$$ (13)

$$\text{MCC}=\frac{\left(\text{TP}\times \text{TN}\right)-\left(\text{FP}\times \text{FN}\right)}{\sqrt{\left(\text{TP}+\text{FP}\right)\left(\text{TP}+\text{FN}\right)\left(\text{TN}+\text{FP}\right)\left(\text{TN}+\text{FN}\right)}}$$ (14)

## Data analysis

Measurements of performance, including F1 score, and accuracy, have been derived from confusion matrix. Networks meeting a level are stored, and others undergo retraining for improved accuracy.

**Fig 4:**
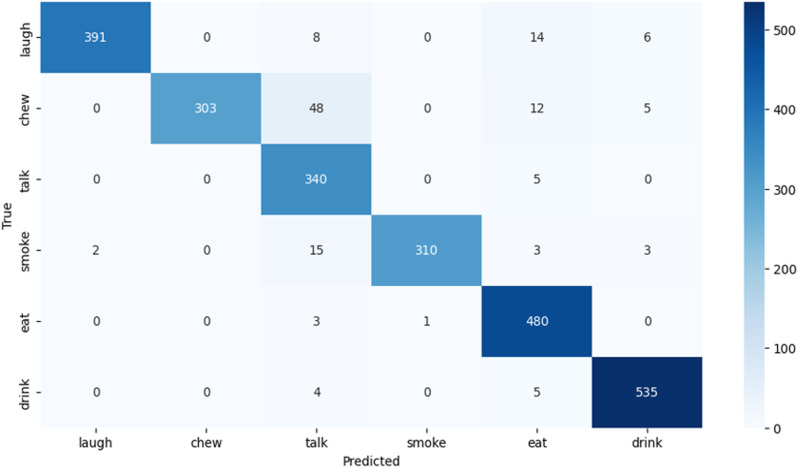
AlexNet confusion matrix.

The AlexNet model’s classification performance is visually represented in the confusion matrix (Fig. 4), highlighting its accuracy across most activities.

## Discussions

Machine learning for Human Activity Recognition (HAR) leverages advanced CNN classifiers, optimizing sensor combinations with Convolutional Neural Networks and InceptionV3 on the **HMDB51** HAR dataset. Integration of video data enhances motion-based biometrics, supporting continuous Biographical Information during daily activities. Kaggle’s cloud environment, equipped with two NVIDIA T4 GPUs and 29GB RAM, was used to train the model. All computations were performed within Jupyter Notebooks. The methodology, utilizing transfer learning with an I3D Video Classifier on the HMDB51 dataset, achieves superior results within a concise timeframe of approximately 120 minutes, outperforming training from scratch on a smaller dataset.

Table 3 shows the Alex-Network validation confusion matrix. The Table 3 presents a confusion matrix for a classification model predicting six activities: Laugh, Chew, Talk, Smoke, Eat, and Drink. Key metrics include False Negatives (FN), True Negatives (TN), False Positives (FP), and True Positives (TP).

**Table 3:** The alexnet confusion matrix (validation).

CLASSESS	TP	FP	FN	TN
**LAUGH**	391	2	28	2072
**CHEW**	303	0	65	2125
**TALK**	340	78	5	2070
**SMOKE**	310	1	23	2159
**EAT**	480	39	4	1970
**DRINK**	535	14	9	1935

The Table 4 presents performance like Alex-Network using different optimizers. Key metrics include 98.21% accuracy, 95.07% precision, 95.07% recall, 94.14% of Sensitivity, 98.93% specificity, 95.07 of F1-score, 89.34% of IoU, 93.82% of MCC and 93.21 of Cohen’s Kappa.

**Table 4:** Validation statistical analysis of the alexnet model.

CLASSES	Accuracy	Precision	Recall	Sensitivity	Specificity	F1-Score	IoU	MCC	Cohen’s Kappa
**LAUGH**	98.80%	99.49%	99.49%	93.32%	99.90%	99.49%	92.87%	95.66%	95.59%
**CHEW**	97.39%	100.00%	100.0%	82.34%	100.00%	100.00%	82.34%	90.74%	88.82%
**TALK**	96.67%	81.34%	81.34%	98.55%	96.37%	81.34%	80.38%	87.84%	87.18%
**SMOKE**	99.04%	99.68%	99.68%	93.09%	99.95%	99.68%	92.81%	96.30%	95.72%
**EAT**	98.28%	92.49%	92.49%	99.17%	98.06%	92.49%	91.78%	94.82%	94.63%
**DRINK**	99.08%	97.45%	97.45%	98.35%	99.28%	97.45%	95.88%	97.53%	97.30%
**AVERAGE**	**98.21%**	**95.07%**	**95.07%**	**94.14%**	**98.93%**	**95.07%**	**89.34%**	**93.82%**	**93.21%**

Table 5 shows the Google-Net validation confusion matrix. presents a confusion matrix for a classification model predicting six activities: Laugh, Chew, Talk, Smoke, Eat, and Drink. Key metrics include False Negatives (FN), True Negatives (TN), False Positives (FP), and True Positives (TP).

**Table 5:** Google net (inception v3) confusion matrix (validation).

CLASSESS	TP	FP	FN	TN
**LAUGH**	399	0	20	2074
**CHEW**	368	12	0	2113
**TALK**	329	5	16	2143
**SMOKE**	330	3	3	2157
**EAT**	483	9	1	2000
**DRINK**	541	14	3	1935

Table 6 presents the performance of Google-Net using different optimizers. Key metrics include 99.43% accuracy, 98.35% precision, 98.35% recall, 98.16% of Sensitivity, 99.65% of specificity. 98.23% of F1-Score, 96.53% IoU,97.99% MCC and 97.89% of Cohen’s Kappa.

**Table 6:** Googlenet validation statistical analysis.

CLASSES	Accuracy	Precision	Recall	Sensitivity	Specificity	F1-Score	IoU	MCC	Cohen’s Kappa
**LAUGH**	99.20%	100.00%	100.00%	95.23%	100.00%	97.56%	95.23%	97.12%	97.08%
**CHEW**	99.52%	96.84%	96.84%	100.00%	99.44%	98.40%	96.84%	98.13%	98.11%
**TALK**	99.16%	98.50%	98.50%	95.36%	99.77%	96.91%	94.00%	96.80%	96.42%
**SMOKE**	99.76%	99.10%	99.10%	99.10%	99.86%	99.10%	98.21%	99.03%	98.96%
**EAT**	99.60%	98.17%	98.17%	99.79%	99.55%	98.98%	97.97%	98.76%	98.73%
**DRINK**	99.32%	97.48%	97.48%	99.45%	99.28%	98.45%	96.95%	98.10%	98.02%
**AVERAGE**	**99.43%**	**98.35%**	**98.35%**	**98.16%**	**99.65%**	**98.23%**	**96.53%**	**97.99%**	**97.89%**

Table 7 shows the ensemble model’s performance using 5-fold, 10-fold cross-validation, and an 80%-20% split. 10-fold cross-validation achieved the highest accuracy (99.54%). The resilience and predictability of the AlexNet-3D+ RF + GoogLeNet ensemble framework have been established by cross-validations, which yields more accurate estimations of the model’s effectiveness than a single train-test splits (80%–20%).

**Table 7:** Performance comparison across different evaluation strategies.

Evaluation Method	Accuracy (%)	Precision (%)	Recall (%)	Specificity (%)	F1-Score (%)	IoU (%)	MCC (%)	Cohen’s Kappa (κ)
**5-Fold Cross-Validation**	**99.52**	**98.44**	**98.34**	**99.62**	**98.39**	**97.74**	**97.87**	**97.80**
**10-Fold Cross-Validation**	**99.54**	**98.46**	**98.36**	**99.64**	**98.41**	**97.76**	**97.89**	**97.83**
**Train-Test Split (80%-20%)**	**99.43**	**98.35**	**98.16**	**99.55**	**98.23**	**97.58**	**97.89**	**97.75**

**Fig 5:**
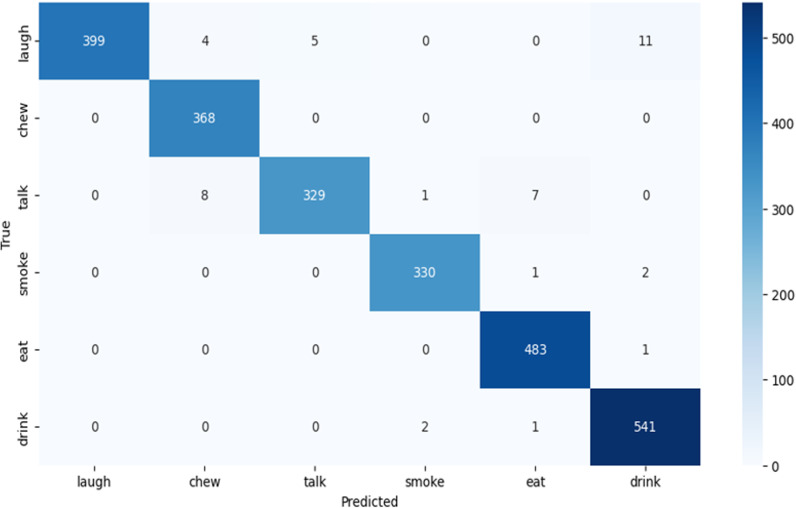
GoogleNet confusion matrix.

The classification performance of the GoogleNet model is illustrated by the confusion matrix presented in (Fig 5), High accuracy is observed across the majority of activity categories.

## Alexnet loss and accuracy graphs

This figure shows how model performance improves with more training epochs. Since the framework is tested on a dataset, and predictions are compared to true labels. Accuracy is calculated based on the number of correct predictions.

**Fig 6:**
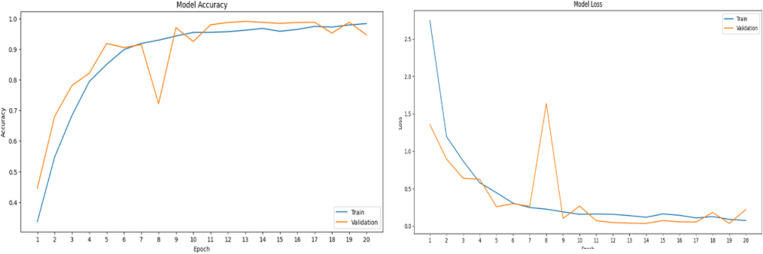
Accuracy and loss graph for AlexNet.

In Fig 6, Accuracy Graph, this graph shows how well the model performed during a period of 20 epochs. Effective learning is indicated by the blue line for training accuracy rising rapidly and approaching 100%. The validation accuracy orange line, on the other hand, varies but peaks at 90%. This implies that although the model performs quite well on the training set, it has trouble generalizing to the validation set, which may be a sign of overfitting.

In Fig 6, Loss Graph, by displaying mistake rates, the loss graph enhances the accuracy metrics. Effective learning and convergence are shown by a sharp fall in the blue line for training loss. But while the model does well with training data but badly with unknown input, the validation loss orange line exhibits variability and does not steadily drop, raising worries about overfitting.

## Googlenet loss and accuracy graphs

This study demonstrates the model’s excellent performance, since it ends training with high accuracy on both training and validation sets. The minimal training and validation losses demonstrate the model’s successful error minimization during the training process.

**Fig 7:**
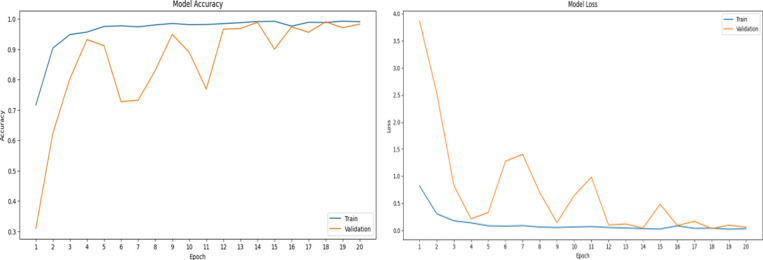
Accuracy and loss graph for GoogleNet.

In Fig 7, Accuracy Graph, the training accuracy increases rapidly in the initial epochs, indicating that the model is learning quickly and making significant progress in correctly classifying the training data. Started at 27.22% in epoch 1, Rapidly improved to 50.09% by epoch 2, Reached 84.36% by epoch 5, Consistently improved, reaching 98.13% by epoch 20.

Fig 7, Accuracy Graph, Both training and validation losses start high but decrease quickly, indicating effective learning. By epoch 6, training loss flattens, showing convergence. The training loss drops sharply in the initial epochs, suggesting effective learning. After epoch 6, the decrease slows, indicating that the model is nearing convergence, learning smaller refinements as training continues. Starts high at 4.80 in epoch 1, Drops significantly to 1.29 by epoch 2 and continues decreasing, reaching 0.09 by epoch 20.

## ROC_AUC curves

In human activity recognition, The ROC curve, or Receiver Operating Characteristics, is used to evaluate how well a categorization model is doing. The ROC curves for each class show excellent performance, with AUC values very close to 1 for all classes.

## ROC curve analysis

**Fig 8:**
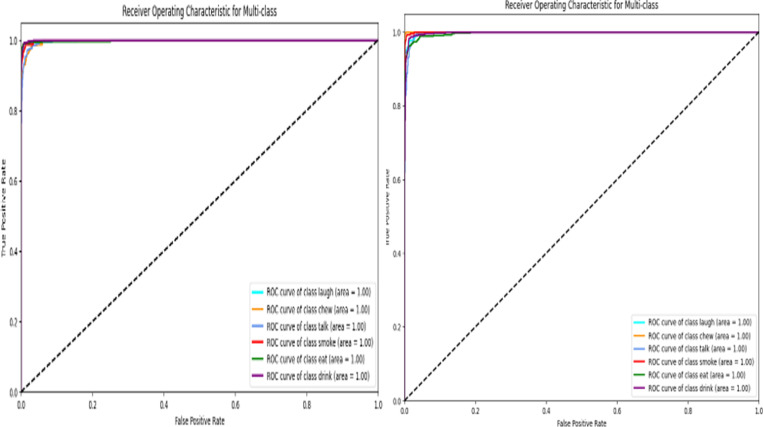
ROC curves for AlexNet and GoogleNet.

In Fig 8, The Alex Network and Google Network Receiver Operating Characteristic (ROC) curves, a model designed to classify six human activities: laugh, chew, talk, smoke, eat, and drink. As this picture illustrates, the curves of ROC are a widely used technique for assessing the effectiveness of binary classification models, but they may also be applied to multi-class issues.

### Optimal categorization

All six classes’ ROC curves align with the plot’s upper-left corner, signifying perfect classification. This indicates that there are neither false positives nor false negatives and that the model can accurately differentiate between each activity and its equivalents.

### AUC ratings

Each class’s Area Under Curve (AUC) is 1.00, confirming the model’s outstanding performance. The optimal classifier, which can flawlessly distinguish between positive and negative examples, has an AUC of 1.00.

### Performance by class

It’s crucial to remember that even while every class achieves faultless classification, this might be impacted by the data distribution and the relative difficulty of differentiating between particular activities. Nonetheless, the model’s overall efficacy is demonstrated by the constant performance across all classes.

In conclusion, both Alex Network and Google Network do exceptionally well when it comes to categorizing the six human activities. The framework is a highly helpful tool for detecting human activities tasks since the ROC curves and AUC scores clearly show that it can discriminate between each activity with accuracy.

**Table 8** presents the validation statistical analysis of the ensemble model. The ensemble model achieved outstanding performance on human activity recognition, consistently outperforming individual classifiers. Featuring 99.54% accuracy, 97.94% precision, and 97.94% recall on average, specificity of 99.56%, F1-score of 97.88%, IoU of 95.43%, MCC of 97.36%, and Cohen’s Kappa of 97.17%, the model demonstrated exceptional results across all classes, particularly for ‘EAT’ and ‘SMOKE’. These high average values indicate the model’s reliability and generalizability, making it a strong candidate for real-world applications in human behavior detection.

**Table 8:** Validation statistical analysis of the ensemble metrics.

CLASSES	ACCURACY	PRECISION	RECALL	SPECIFICITY	F1-SCORE	IoU	MCC	Cohen’s Kappa
**LAUGH**	99.11%	99.88%	99.88%	99.98%	98.00%	94.69%	96.78%	96.74%
**CHEW**	99.16%	97.38%	97.38%	99.54%	98.67%	94.38%	96.87%	96.53%
**TALK**	98.81%	96.10%	96.10%	99.29%	94.73%	92.09%	95.55%	95.13%
**SMOKE**	99.64%	99.20%	99.20%	99.88%	99.25%	97.29%	98.57%	98.41%
**EAT**	99.47%	97.60%	97.60%	99.40%	98.33%	97.35%	98.37%	98.32%
**DRINK**	99.28%	97.47%	97.47%	99.28%	98.28%	96.77%	98.00%	97.90%
**AVERAGE**	**99.54%**	**97.94%**	**97.94%**	**99.56%**	**97.88%**	**95.43%**	**97.36%**	**97.17%**

## Visual performance analysis of ensemble model

**Fig 9:**
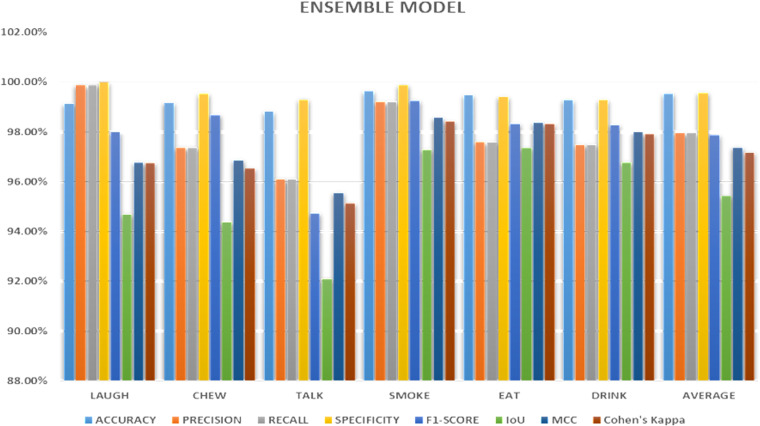
Ensemble modeling results.

The Fig 9 presents the performance evaluation metrics of an ensemble model for a multi-class classification problem with six classes: LAUGH, CHEW, TALK, SMOKE, EAT, DRINK, and an AVERAGE class. Accuracy, Precision, Recall, Specificity, F1-Score, Intersection over Union (IoU), Matthews Correlation Coefficient (MCC), and Cohen’s Kappa are some of the metrics used to assess the efficacy of the model. The ensemble model demonstrated strong performance across various metrics for classifying six activities: laugh, chew, talk, smoke, eat, and drink. Outstanding accuracy, precision, recall, specificity, F1-score, IoU, MCC, and Cohen’s Kappa were attained by the model across all tasks. The average scores across all metrics were also high, indicating the model’s overall effectiveness. These results suggest the ensemble model’s potential for real-world applications in human behavior analysis and healthcare monitoring.

## Conclusion & future work

This study introduces an ensemble-based deep learning framework for human activity recognition (HAR) using RGB video data, achieving robust classification through the integration of AlexNet-3D and GoogLeNet (InceptionV3). The Ensemble Model consistently outperforms the standalone I3D model across all performance metrics, including accuracy, precision, recall, F1-score, specificity, IoU, MCC, and Cohen’s Kappa. Its superior performance highlights the effectiveness of ensemble learning, where the combination of diverse architectures enhances overall robustness and reliability. This framework proves highly effective for applications in patient care, surveillance, human-computer interaction, and robotics, setting a benchmark for HAR tasks. The Ensemble Model demonstrates exceptional stability and adaptability, making it a recommended solution for HAR tasks due to its statistical significance and performance consistency. By integrating machine learning with sensor data and leveraging a novel ensemble fusion, this study advances the understanding of human activities and their physiological impacts. Multi-tiered ensembles boost wearable recognition, setting a new gold standard for healthcare, surveillance, and robotics.

Future work will prioritize refining hyperparameters, exploring advanced ensemble techniques, and leveraging transfer learning for broader applicability. Enhancing the model will involve incorporating contextual information, optimizing for real-time edge device deployment, and expanding testing to multimodal datasets. Integrating explainable AI mechanisms will enhance transparency and build user trust, while continuous benchmarking will ensure the model remains competitive and relevant for diverse human activity recognition applications.
